# Long and short sleep durations can affect cognitive function in older adults through the chain mediation effect of ADL and depression: evidence from CHARLS2018

**DOI:** 10.1007/s40520-024-02881-w

**Published:** 2024-11-20

**Authors:** Hao Zou, Lijie Jiang, Yueli Hou, Linlin Zhang, Jianrong Liu

**Affiliations:** 1 The Second Affiliated Hospital of Xi’an Medical University, Xi’an, Shaanxi 710038 China; 2 Department of nursing, school of pharmacy, Changzhou University, Jiangsu, 213164 China

**Keywords:** Chain mediating effect, Activities of daily living, Sleep durations, Cognitive function

## Abstract

**Background:**

Both long and short sleep durations may lead to cognitive decline in the elderly individuals, though the underlying mechanisms remain unclear.

**Aims:**

To explore the mediating mechanism of activities of daily living and depression on different sleep durations and cognitive function in older Chinese older adults.

**Methods:**

This retrospective study used data from 5,899 older adults who completed the 2018 China Health and Retirement Longitudinal Surveys. We used the PROCESS macro in SPSS to determine the chain mediating effect of ADL and depression on the relationship between different sleep durations and cognitive functions.

**Results:**

(1) There were significant correlations among sleep duration, ability to perform ADL, depression, and cognitive function. (2) For sleep durations ≤ 7 h/night or > 7 h/night, ADL and depression play a chain mediating role in the relationship between sleep duration and cognitive function.

**Conclusions:**

Shorter or longer sleep durations were associated with cognitive decline by weakening ADL and worsening depression, which suggests that medical personnel should take action to correct abnormal sleep duration in older adults. Timely treatment of ADL impairment and depression may help prevent cognitive decline.

## Introduction

Senile dementia is an age-related syndrome caused by the degeneration of the brain’s nervous system, and manifests as a variety of cognitive dysfunctions, including losses of memory, thinking ability, and problem solving ability and changes in speech [1, 2]. In 2018, the global population of individuals living with dementia was estimated to be 50 million, and this number is projected to triple by 2050 [3]. China has approximately 9.5 million patients with dementia, ranking first in the world [4]. Due to the incurability of neurodegenerative diseases, studying the early prevention of dementia has practical value [5]. Cognitive decline is a typical early manifestation of dementia, and the early mitigation of risk factors for cognitive decline is of great significance for avoiding dementia [1].

Sleep plays an important role in brain maturation as well as the development and maintenance of cognitive functions [2]. However, more than 30% of older adults report various issues with sleep [6]. Abnormal sleep durations in the form of shortened sleep or lengthened periods of sleep are common in older adults, with approximately 12% and 22% of older adults experiencing short (< 6 h) and long (≥ 9 h) sleep durations, respectively [7]. Both types of abnormal sleep durations increase the risk of cognitive decline [8]. Studies have shown that an abnormal sleep duration can cause an increase in C-reactive protein (CRP) and interleukin-6 (IL-6) levels, which play a role in regulating inflammatory processes, and elevated levels of inflammatory cytokines can lead to decreased cognitive function [9]. Prolonged sleep deprivation may lead to amyloid-β deposits in the brain, which increase the risk of cognitive decline [9].

A previous study reported that the rate of depressive symptoms detection in older Chinese adults was 31.0% [10], and depression was the most common neuropsychiatric symptom in patients with mild cognitive impairment (26.1%) [11]. Depression also significantly predicts cognitive decline, with greater severity associated with faster cognitive decline [12, 13]. Current studies suggest that depression may affect cognitive function via vascular damage, stress hormone pathway activation, and decrease in brain-derived neurotrophic factor (BDNF) and serotonin levels. Additionally, sleep duration can affect depression [14]. A meta-analysis revealed a U-shaped relationship between sleep duration and depression risk, with both shorter and longer sleep durations associated with an increased risk of depression compared with a 7-hour sleep duration [15].

Activities of daily living (ADL) are a series of basic activities that must be performed in daily life, and included basic ADL (BADL) and instrumental ADL (IADL). BADL include basic self-care abilities (e.g., bathing, dressing, and eating), and IADL include the individual’ s adaptation to the surrounding environment (e.g., cooking, shopping, and doing housework) [16]. A survey of 10,148 older adults in China revealed that 26.56% experienced ADL impairment [17], which is often accompanied by cognitive function decline and depression [16]. An impaired ability to perform ADL restricts the daily life and social activities of older adults to a certain extent, and it directly affects depressive symptoms and cognitive function decline in older adults [18, 19]. Furthermore, long and short sleep durations are associated with an increased risk of impaired ability to perform ADL [20]. A Chinese study revealed that CRP levels are positively associated with the risk of ADL disability in elderly individuals, and abnormal sleep duration can lead to an increase in CRP, suggesting that abnormal sleep duration may increase the risk of ADL impairment via inflammation [21]. A study conducted in the United States reported that not attaining optimal sleep durations (7–8 h/weeknight) is associated with an impaired ability to perform ADL in older adults and that a long sleep duration is associated with fatigue and a lack of physical activity; moreover, older adults who sleep longer are more likely to be limited in activities that require more physical exertion (such as bathing, lower extremity mobility activities, and general physical activities)[22].

These results revealed a correlation between sleep durations, ADL, depression and cognitive function, Changes in sleep duration may lead to changes in ADL and depression, thus affecting cognitive function. The health ecological model proposes that individual health is the result of interdependence and interaction among individual factors, psychological and behavioral factors, and factors related to the living environment [23]. In the context of an aging population, the maintenance of good cognitive function is an important indicator for evaluating health, and may be the result of the interaction between individual factors (i.e., ADL) and psychological and behavioral factors (depression and sleep duration). However, until now, no studies have reported an association between sleep duration, ADL, depression, and cognitive function, and no studies have explored the complex mechanisms of such an association.

Therefore, after reviewing the correlations among the above four variables, this study hypothesized that ADL and depression play a chain mediating role in the relationship between sleep duration and cognitive function. To explore this, the underlying mechanisms of action of different sleep durations was analyzed, which may provide insight into how cognitive function decline can be prevented in older adults with different sleep duration characteristics.

## Materials and methods

### Population

Data were obtained from the China Health and Retirement Longitudinal Surveys (CHARLS), which is hosted by the National Development Research Institute of Peking University. CHARLS is a large-scale interdisciplinary longitudinal national survey of Chinese residents aged 45 years and above. The baseline survey was conducted in 2011, and included 17,706 middle-aged and older Chinese adults (> 45 years of age) in 28 provinces (autonomous regions and municipalities directly under the Central Government), 150 counties, 450 communities (villages) and 10,257 households. CHARLS follow-up occurs every 2 years (2011: first wave, 2013: second wave, 2015: third wave, 2018: fourth wave) [24].

We used data from the CHARLS 2018 (fourth wave), which included 19,744 participants. For the current analysis, eligible participants had completed demographic information and provided scores for cognitive function, depression, ADL, and sleep duration. Ultimately, 5899 older adults (≥ 60 years of age) were included in this study. Ethical approval for the collection of data from the CHARLS was obtained from the Institutional Review Board of Peking University (IRB00001052-11015).

### Measurements

#### Demographic characteristics

Sociodemographic variables such as age (continuous), sex (male, female), education (illiteracy, primary, middle, high or above), marriage (married, unmarried/divorced/widowed), number of chronic diseases (continuous), and wage income (no, yes) were extracted from the dataset and included as covariated in the subsequent mediation analysis to control for potential confounding factors.

#### Cognitive function

The Chinese version of the Mini-Mental State Examination scale (MMSE) was used to assess cognitive function. The MMSE comprises a total of 30 items, and includes five dimensions (orientation, registration, attention and calculation, recall, and language). Each correct answer is awarded 1 point, and the total score is between 0 and 30 points, with higher scores indicating better cognitive function [25].

#### Depression

The 10-item Center for Epidemiological Studies Depression Scale (CESD-10) was used to assess depression. The 10 questions assessed the participants’ feelings and behaviors over the past week (e.g., “I was bothered by things that don’t usually bother me”, “I had trouble keeping my mind on what I was doing”, and “I felt depressed”, etc.). The score of each item on the CESD-10 ranges from 0 (< 1 day) ~ 3 (5 ~ 7 days), with a total score ranging from 0 ~ 30. Higher scores are indicative of more severe depressive symptoms [26].

#### Sleep duration

Self-reported nocturnal sleep duration data were obtained via the following question: “During the past month, how many hours of actual sleep did you get at night (average hours for one night)?”. The results of a prospective study in China revealed that individuals with 6–7 h of nocturnal sleep had the lowest risk of cognitive impairment [27]; The results from two previous meta-analyses also revealed that individuals who sleep 7 h per night have a lower risk of MCI/dementia, whereas those who fall below or exceed the 7-h threshold have a greater risk of MCI/ dementia [28, 29]. In a 25-year follow-up study in the UK, individuals who slept less than 7 h per night (short sleep) had an increased risk of developing dementia [30]. These studies revealed a nonlinear relationship between sleep duration and cognitive impairment. Therefore, we considered 7 h of sleep per night as the threshold and divided the reported sleep durations into short sleep (≤ 7 h/night) and long sleep (> 7 h/night).

### ADL

The total BADL and IADL scores are used to comprehensively assess ADL. The BADL scale includes 6 items: (1) dressing; (2) bathing; (3) eating; (4) getting into or out of bed; (5) using the toilet; and (6) defecation. The IADL scale includes 5 items: (1) doing household chores; (2) cooking; (3) shopping; (4) taking medications; and (5) managing money. These 11 items were used to assessed ADL in older adults. All of the items are rated from 0 (completely independent) to 3 (completely dependent), with a total score of 0–33. Higher scores are indicative of worse performance in ADL [16, 31].

### Statistical analysis

In this study, we divided the participants into two groups (short and long sleep), explored the correlations between the variables in each group, and constructed a chain mediation models separately.

SPSS version 22.0 was used for the data analysis. Categorical variables are presented as frequencies, and continuous variables that do not conform to a normal distribution are presented as medians and quartile spacings [M (p25, p75)]. Spearman correlation analyses were used to determine the associations between variables. Model 6 in the PROCESS macro (http://www.afhayes.com) compiled by Hayes was used to test the chain intermediary effect. The Bootstrap 95% confidence interval (CI) was used to test whether regression coefficients were significant for estimating the chain mediation effect from 5000 samples. If the 95% CI did not include 0, the indirect effect was considered statistically significant [32].

## Results

### Sociodemographic characteristics

A total of 5899 older adults were included in this study. The median age of the participants was 67.0 years. Most participants had an average sleep duration ≤ 7 h per night (74.5%), were female (53.6%), were married (80.7%), were illiterate (50.5%), and had no current wage income (89.0%) (Table 1).

**Table 1:** Sociodemographic characteristics of participants (*N* = 5899)

Variables	Characteristics	Participants M (p25, p75)/ *N*(%)
Age		67.0 (64.0, 72.0)
No. of Chronic Diseases		1.0 (0.0, 1.0)
Sleep duration	≤ 7 h/night	4392 (74.5)
	>7 h/night	1507 (25.5)
Sex	Male	2735 (46.4)
	Female	3164 (53.6)
Marital status	Married	4763 (80.7)
	Unmarried/Divorced/Widowed	1136 (19.3)
Education level	Illiteracy	2981 (50.5)
	Primary school	1381 (23.4)
	Middle school	958 (16.2)
	High school or above	579 (9.8)
Wage income	No	5252 (89.0)
	Yes	647 (11.0)

Abbreviations: N, numbers; M(P25, P75), median and interquartile range; No. of Chronic Diseases, number of chronic diseases

### Correlation analysis

In the group with an average sleep duration of ≤ 7 h/night, the sleep duration was negatively correlated with ADL (*r*=-0.230, *P* < 0.01) and depression (*r*=-0.341, *P* < 0.01) and positively correlated with cognitive function (*r* = 0.208, *P* < 0.01); ADL were positively correlated with depression (*r* = 0.367, *P* < 0.01) and negatively correlated with cognitive function (*r*=-0.241, *P* < 0.01); and finally, depression was negatively correlated with cognitive function (*r*=-0.275, *P* < 0.01).

In the group with an average sleep duration > 7 h/night, the sleep duration was positively correlated with ADL (*r* = 0.124, *P* < 0.01) and depression (*r* = 0.052, *P* < 0.05), and negatively correlated with cognitive function (*r*=-0.200, *P* < 0.01); ADL were positively correlated with depression (*r* = 0.283, *P* < 0.01), and negatively correlated with cognitive function (*r*=-0.212, *P* < 0.01). Finally, depression was negatively correlated with cognitive function (*r*=-0.209, *P* < 0.01) (Table 2).

**Table 2:** Statistical description and correlation analysis results

		M(p25, p75)	ADL	DP	SD	CF
SD ≤ 7 h/night	ADL	0.0 (0.0, 3.0)	1			
	DP	9.0 (5.0, 15.0)	0.367^**^	1		
	SD	5.0 (4.0, 6.0)	-0.230^**^	-0.341^**^	1	
	CF	21.0 (17.0, 25.0)	-0.241^**^	-0.275^**^	0.208^**^	1
SD>7 h/night	ADL	0.0 (0.0, 2.0)	1			
	DP	7.0 (3.0, 13.0)	0.283^**^	1		
	SD	8.0 (8.0, 9.0)	0.124^**^	0.052^*^	1	
	CF	21.0 (16.0, 24.0)	-0.212^**^	-0.209^**^	-0.200^**^	1

Note: *P < 0.05, **P < 0.01.

Abbreviations: ADL, activities of daily living; DP, depression; SD, sleep duration; CF, cognitive function; M(P25, P75), median and interquartile range

### Chain mediation analysis

Model 6 in the PROCESS macro compiled by Hayes was used to test the chain mediating effect of ADL and depression on the relationship between sleep duration and cognitive function. In the short sleep group (≤ 7 h/night), the sleep duration had a direct positive predictive effect on cognitive function (β = 0.3153, *P* < 0.01). Sleep duration negatively predicted ADL (β=-0.5002, *P* < 0.01) and depression (β=-1.1734, *P* < 0.05). ADL negatively predicted cognitive function (β=-0.1530, *P* < 0.01) and positively predicted depression (β = 0.4639, *P* < 0.01). Finally, the depression negatively predicted the cognitive function (β=-0.0691, *P* < 0.01) (Table 3).

In the long sleep group (> 7 h/night), the sleep duration had a direct negative predictive effect on cognitive function (β=-0.6707, *P* < 0.01) and positively predicted ADL (β = 0.4221, *P* < 0.01). ADL negatively predicted cognitive function (β=-0.1229, *P* < 0.01) and positively predicted depression (β = 0.4099, *P* < 0.01). Finally, the depression negatively predicted the cognitive function (β=-0.1081, *P* < 0.01).

**Table 3:** Chain mediation model

	Outcome variable	Predictor variable	*R*	*R* ^2^	F	β	t
SD ≤ 7 h/night	ADL	SD	0.2996	0.0897	61.7473	-0.5002	-13.0156^**^
	DP	SD	0.4884	0.2386	171.6623	-1.1734	-18.7675^*^
		ADL				0.4639	19.2424^**^
	CF	SD	0.5928	0.3514	263.7477	0.3153	6.7226^**^
		ADL				-0.1530	-8.4407^**^
		DP				-0.0691	-6.3358^**^
SD > 7 h/night	ADL	SD	0.2545	0.0647	14.8254	0.4221	4.9887^**^
	DP	SD	0.3317	0.1100	23.1419	0.1419	0.9924
		ADL				0.4099	9.4703^**^
	CF	SD	0.5614	0.3152	76.5689	-0.6707	-6.3926^**^
		ADL				-0.1229	-3.7600^**^
		DP				-0.1081	-5.7001^**^

Note: Controlling for age, sex, number of chronic diseases, marital status, education level and wage income, *P < 0.05,**P < 0.01.

Abbreviations: ADL, activities of daily living; DP, depression; CF, cognitive function; SD, sleep duration

Bootstrap analysis was used to further test the chain mediation effect (Table 4). In the short sleep group (≤ 7 h/night), the direct effect of sleep duration on cognitive function was significant, with an effect size of 0.3153, (95% CI: (0.2234, 0.4073)). The effect sizes of indirect effect 1 (Sleep duration→ADL→Cognitive function), indirect effect 2 (Sleep duration→Depression→Cognitive function), and indirect effect 3 (Sleep duration→ADL→Depression→Cognitive function) were 0.0765, 0.0811 and 0.0160, respectively. Their 95%CIs were (0.055, 0.0991), (0.0552, 0.1085), and (0.0104, 0.0224), respectively, which did not include 0, indicating that these paths were statistically significant.

**Table 4:** Bootstrap analysis

	Path	Effect size	Standard error	Boot 95% CI	
				LL	UL
Sleep duration ≤ 7 h/night	Direct effect	0.3153	0.0469	0.2234	0.4073
	Indirect effect 1	0.0765	0.0111	0.0551	0.0991
	Indirect effect 2	0.0811	0.0135	0.0552	0.1085
	Indirect effect 3	0.0160	0.0030	0.0104	0.0224
	Total mediation effect	0.1736	0.0180	0.1383	0.2100
Sleep duration > 7 h/night	Direct effect	-0.6707	0.1049	-0.8765	-0.4649
	Indirect effect 1	-0.0519	0.0203	-0.0975	-0.0188
	Indirect effect 2	-0.0153	0.0156	-0.0487	0.0129
	Indirect effect 3	-0.0187	0.0065	-0.0334	-0.0075
	Total mediation effect	-0.0859	0.0273	-0.1441	-0.0373

Note: Direct effect: Sleep duration→Cognitive function

Indirect effect 1: Sleep duration→ADL→Cognitive function

Indirect effect 2: Sleep duration→Depression→Cognitive function

Indirect effect 3: Sleep duration→ADL→Depression→Cognitive function

Similarly, in the long sleep group with an average sleep duration (> 7 h/night), the direct effect of sleep duration, the mediating effect of ADL (indirect effect 1), and the chain mediating effect of ADL and depression (indirect effect 3) were significant. effect sizes were − 0.6707, -0.0519, and − 0.0187, respectively, and the 95% CIs were (-0.8765, -0.4649), (-0.0975, -0.0188), and (-0.0334, -0.0075), respectively. However, the mediating effect of depression was not significant (indirect effect 2, 95% CI: (-0.0487, 0.0129)).

**Fig. 1:**
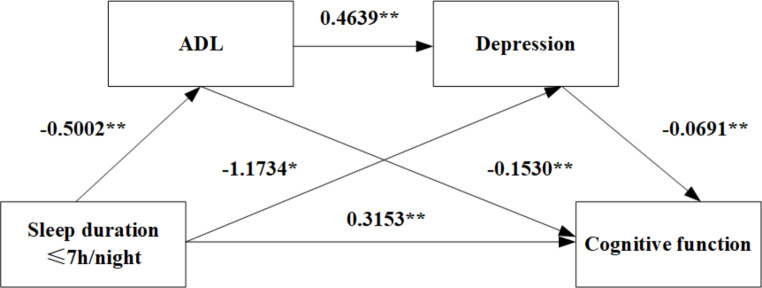
The chain mediation model for ADL, depression, sleep duration (≤ 7 h/night) and cognitive function, *P < 0.05, **P<0.01

**Fig. 2:**
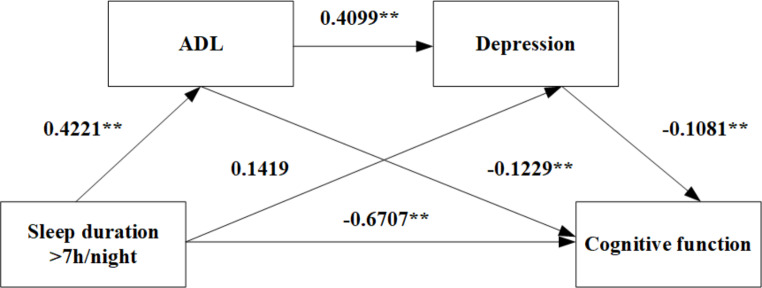
The chain mediation model for ADL, depression, sleep duration (> 7 h/night) and cognitive function, **P<0.01

Therefore, these results support the previous hypothesis that ADL and depression act as chain mediators between sleep duration and cognitive function, both in the short and long sleep groups (Figs. 1 and 2).

## Discussion

### The mediating effect of ADL on the relationship between sleep duration and cognitive function

This study revealed that ADL play a partial mediating role between sleep duration and cognitive function, though the mechanism influencing this effect differs based on the sleep duration. In the group with an average sleep duration ≤ 7 h/night, the sleep duration negatively predicted ADL; therefore, a shorter sleep duration was associated with worse ADL. In the group with an average sleep duration > 7 h/night, the sleep duration positively predicted ADL; therefore, longer sleep durations were associated with worse ADL, thers results were consistent with previous research [19]. Studies have shown that short or long sleep durations can increase the risk of cardiovascular events, osteoporosis, and stroke in older adults, which all lead to functional limitations [33]. In addition, abnormal sleep duration may lead to poor sleep quality, which causes mental fatigue, leads to physical dysfunction, and subsequently affects the ability to perform ADL among elderly individuals [34].

The current study also revealed that, ADL had a negative relationship with cognitive function in both sleep duration groups; therefore, worse ADL were associated with worse cognitive function. Impaired ADL may reduce the lifespan and range of activities performed by older adults and reduce the opportunity to communicate externally. These effects reduce the amount of effective stimulation required by the human brain, thus accelerating declines in cognitive function [35]. Moreover, a decline in cognitive function diminishes the ability to complete some complex self-care activities, further weakening ADL, thus forming a negative feedback loop [35]. Both insufficient and excessive sleep accelerate cognitive decline in older adults by weakening ADL.

### The mediating effect of depression on the relationship between sleep duration and cognitive function

This study revealed that in the group with an average sleep duration ≤ 7 h/night, sleep duration further affected cognitive function through the mediating effect of depression. Mechanistically, shorter sleep durations lead to more severe depression symptoms and worsened cognitive function. One meta-analysis reported that the combined prevalence of sleep disorders and depression was 10.6% in the elderly population, with sleep disorders representing both a risk factor for the development of depression and a precursor to the onset of depression. Sleep disorders mainly include insomnia, poor sleep quality and sleep complaints, which may lead to reduced sleep duration [36, 37]. Reduced sleep duration may lead to daytime fatigue, which may lead to an increase in negative experiences and emotions, which may promote the development of depression [38]. Studies have shown that older adults with depressive symptoms engage in fewer social activities and are less capable of thinking and concentrating, while those with depression are significantly less capable in all cognitive areas [39].

### The chain mediating effect of ADL and depression on the relationship between sleep duration and cognitive function

This study revealed that, in both groups, ADL and depression played a chain mediating role between sleep duration and cognitive function and that ADL were positively associated with depression (i.e., worse ADL were associated with more severe depression). Poorer ADL indicate that individuals are engaging in less self-care and have a lower ability to perform basic daily activities (e.g., bathing, dressing, and shopping) independently, which can lead to feelings of hopelessness or worthlessness because of a loss of independent identity [40]. Older adults with ADL impairment need long-term care from family members or other caregivers, which may create tension in these relationships. Moreover, a loss of the ability to engage in self-care, significantly reduces interpersonal communication, the frequency of participation in social activities, and an inability to effectively deal with negative emotions, resulting in a psychological environment in which depression can develop [40, 41]. In turn, depression accelerates cognitive decline. A previous study reported that depression leads to increased circulation of glucocorticoids, which in turn leads to atrophy of the hippocampus, the center of the episodic memory network that plays an important role in memory formation, storage, retrieval and the processing of emotions [42, 43].

In conclusion, this study revealed that the mechanism of the effect of sleep duration on cognitive function differs depending on the sleep duration. In the group with an average sleep duration ≤ 7 h/night, the shorter sleep duration was associated with a worsened ability to perform ADL and more serious depression, leading to a decline in cognitive function. In the group with an average sleep duration > 7 h/night, the longer sleep duration was associated with a worsened ability to perform ADL, and more severe depression, ultimately leading to a decline in cognitive function. These two different mechanisms indicate that sleep duration may have a U-shaped relationship with ADL and cognitive function; that is, both short sleep and long sleep durations may lead to ADL impairment and cognitive function decline. These mechanisms both lead to depression by weakening ADL and indirectly leading to cognitive function decline. However, in the group with an average sleep duration > 7 h/night, sleep duration did not affect cognitive function through the mediating effect of depression, but through the chain mediating effect of ADL and depression. These results suggest that for older adults with long sleep durations, depression is more likely to be caused by impaired ADL, indicating that healthcare professionals should carefully assess ADL in older adults with long sleep durations. Furthermore, appropriate measures should be taken to improve the ADL with the aim of improving depressive symptoms and preventing cognitive decline. In conclusion, the results of this study suggest that healthcare providers should discuss the importance of keeping sleep within the normal range (6–7 h/night) with older adults to maintain cognitive function, carefully assess ADL and depressive symptoms in older adults with abnormal sleep durations, and promptly treat ADL impairment and depression to avoid cognitive decline.

### Limitations

First, the variables in this study, especially sleep duration, represent subjective measures. This study only considers the sleep status of older adults in the month prior to the survey, and the sleep duration was self-reported. Participants with poor cognitive function may have misreported their experiences; therefore, future studies with more objective measures or that incorporate reports from family members are necessary to obtain more accurate sleep durations. Second, to obtain the complete data, some older adults whose data were incomplete were excluded, which may have biased the results. Finally, this study examined only the horizontal relationships between variables, as it is cross-sectional, and the causal relationships among them remain insufficiently explained. Longitudinal studies are needed to explore and verify the potential U-shaped relationship between sleep duration, ADL, and cognitive function. Despite these limitations, this study has several advantages. This is the first study to use large-scale data to explore the mechanisms of different sleep durations on cognitive function. The chain mediation between ADL and depression provides new insight into the mechanism by which sleep duration affects cognitive function and provides a theoretical basis for preventing cognitive function decline in older adults.

## Data Availability

CHARLS is a public open access dataset. Down link: http://charls.pku.edu.cn/en.
